# Melittin from *Apis florea* Venom as a Promising Therapeutic Agent for Skin Cancer Treatment

**DOI:** 10.3390/antibiotics9080517

**Published:** 2020-08-14

**Authors:** Sirikwan Sangboonruang, Kuntida Kitidee, Panuwan Chantawannakul, Khajornsak Tragoolpua, Yingmanee Tragoolpua

**Affiliations:** 1 Biotechnology Section, Graduate School, Chiang Mai University, Chiang Mai 50200, Thailand; sirikwan_s@cmu.ac.th; 2 Center for Research and Innovation, Faculty of Medical Technology, Mahidol University, Salaya, Nakhon Pathom 73170, Thailand; kuntida.kit@mahidol.edu; 3 Division of Microbiology, Department of Biology, Faculty of Sciences, Chiang Mai University, Chiang Mai 50200, Thailand; panuwan@gmail.com; 4 Division of Clinical Microbiology, Department of Medical Technology, Faculty of Associated Medical Sciences, Chiang Mai University, Chiang Mai 50200, Thailand; khajornsak.tr@cmu.ac.th; 5 Infectious Diseases Research Unit (IDRU), Faculty of Associated Medical Sciences, Chiang Mai University, Chiang Mai 50200, Thailand; 6 Research Center in Bioresources for Agriculture, Industry, and Medicine, Department of Biology, Faculty of Science, Chiang Mai University, Chiang Mai 50200, Thailand

**Keywords:** melittin, *Apis florea*, malignant melanoma, apoptosis, F-actin, epidermal growth factor receptor

## Abstract

Melittin, a major component found in bee venom, is produced by the *Apis* species of the honey bee. In this study, the effect of melittin derived from *Apis florea* (Mel-AF), which is a wild honey bee species that is indigenous to Thailand, was investigated against human malignant melanoma (A375) cells. In this study, Mel-AF exhibited considerable potential in the anti-proliferative action of A375 cells. Subsequently, the cellular mechanism of Mel-AF that induced cell death was investigated in terms of apoptosis. As a result, gene and protein expression levels, which indicated the activation of cytochrome-c release and caspase-9 expression, eventually triggered the release of the caspase-3 executioner upon Mel-AF. We then determined that apoptosis-mediated cell death was carried out through the intrinsic mitochondrial pathway. Moreover, advanced abilities, including cell motility and invasion, were significantly suppressed. Mel-AF manipulated the actin arrangement via the trapping of stress fibers that were found underneath the membrane, which resulted in the defective actin cytoskeleton organization. Consequently, the expression of EGFR, a binding protein to F-actin, was also found to be suppressed. This outcome strongly supports the effects of Mel-AF in the inhibition of progressive malignant activity through the disruption of actin cytoskeleton-EGFR interaction and the EGFR signaling system. Thus, the findings of our current study indicate the potential usefulness of Mel-AF in cancer treatments as an apoptosis inducer and a potential actin-targeting agent.

## 1. Introduction

Malignant melanoma is the most serious form of skin cancer and originates from melanocyte. It has been associated with rapid progression and metastatic potential. Importantly, the mortality rate of this disease has been increasing worldwide and conventional treatments such as surgery, chemo- and radiation therapy are quite often unsuccessful [1,2]. Besides these hindrances, this cancer is difficult to treat when the metastatic stage has developed. Additionally, it also responds poorly to cytotoxic agents, while being especially resistant to drug-induced apoptosis [1,2,3]. There are limitations associated with existing medications; therefore, the exploration of alternative agents, such as natural compounds or bioactive substances that display active biological properties, potent pharmaceutical efficiency, and a degree of safety with fewer side effects, are considered extremely essential. Currently, various natural products and bioactive compounds have displayed effective activity in inhibiting the growth of many cancer cells on their own or in combination with other agents [4,5]. Significantly, they also can induce cancer cell death via the apoptosis pathway [4], which is the ultimate goal of many chemotherapeutic agents and treatment strategies [6,7]. Therefore, the development of a new effective bioactive agent that could stimulate the apoptotic cell death pathway would be extremely useful.

Bee venom or Apitoxin is a natural toxin produced by the *Apis* species of the honey bee and has been used for many years in “Apitherapy” as a form of traditional medicine to treat pain and a variety of diseases [8,9,10]. This toxin is comprised of a number of biologically active ingredients including peptides (e.g., melittin, apamine, mast-cell-degranulating (MCD) and adolapin), enzymes, amines and other small molecules [10,11]. Nevertheless, the crude extract of bee venom itself contains complex components that can cause undesirable effects. The PLA2 and MCD peptides present in bee venom are the principal allergens and are associated with a range of allergic reactions [8,10,12]. A previous study conducted by Somwongin et al. (2018) has provided supporting data on the irritation properties of the bee venom that was obtained from four honey bees (*A. mellifera*, *A. dorsata*, *A. cerana* and *A. florea*). It was reported that the bee venom extract from *A. mellifera* caused a strong degree of irritation while the bee venom of *A. florea* revealed no signs of irritation [13]. Thus, many research studies have attempted to investigate the optimal applications of bee venom while minimizing any irritable effects. This is often done through the use of melittin, which is the primary active component in bee venom. As melittin is the principal component of bee venom, it constitutes approximately 40–50% of the venom’s dry weight. It is a small linear cationic peptide consisting of 26-amino acids [10,14]. Its action is associated with the electrostatic interaction of negatively charge membranes and causes pore formation to undergo cell membrane disruption [15,16]. The anti-cancer activity and apoptosis induction of bee venom and melittin (*A. mellifera*) has been studied on a number of cancer cells, e.g., human ovarian cancer (SKOV3 and PA-1) cells, human leukemia U937 cells, and human melanoma (A2058) cells [11,14,15,17,18]. The findings reveal the high potential of melittin to be used as an effective agent in cancer therapy. As the melittin peptide sequence is originally derived from *A. mellifera* (European honey bee), the most common honey bee in Apiculture worldwide, its biological and pharmaceutical attributes have been vastly elucidated [14,15,19]. However, it has been reported that certain distinct species of honey bees contain differing amino acid sequences of melittin [20,21]. Moreover, due to the reduced degree of irritation that is associated with the bee venom of *A. florea* [13], the melittin contained in this venom is the focus of this study.

The biological properties of melittin obtained from *A. florea* (dwarf honey bee), an indigenous honey bee of southern and southeastern Asia and the wild honey bee that is commonly found in Thailand, are not fully understood and there have been no published reports focusing on its potential anticancer activity. In order to gain a better understanding of the cellular mechanisms of melittin obtained from *A. florea* (Mel-AF), the anti-cancer effect and apoptotic cell death induction on human malignant melanoma (A375) cells by Mel-AF was investigated in comparison with *A. mellifera* (Mel-AM).

Our observations revealed the greatest activity in terms of the anti-proliferative, anti-migration and anti-invasion capabilities of Mel-AF against A375 cells, along with the possibility that it could serve as a mechanism for growth inhibition. These were all representative of its aggressive abilities toward cancer cells. Taken together, we suggest that Mel-AF could be an attractive agent for use in alternative medicine and in further high value applications, such as in combination therapy and nanomedicine for malignant melanoma and other human cancers.

## 2. Results

### 2.1. Protein Analysis of Crude Bee Venom Extracted from A. florea

**Figure 1:**
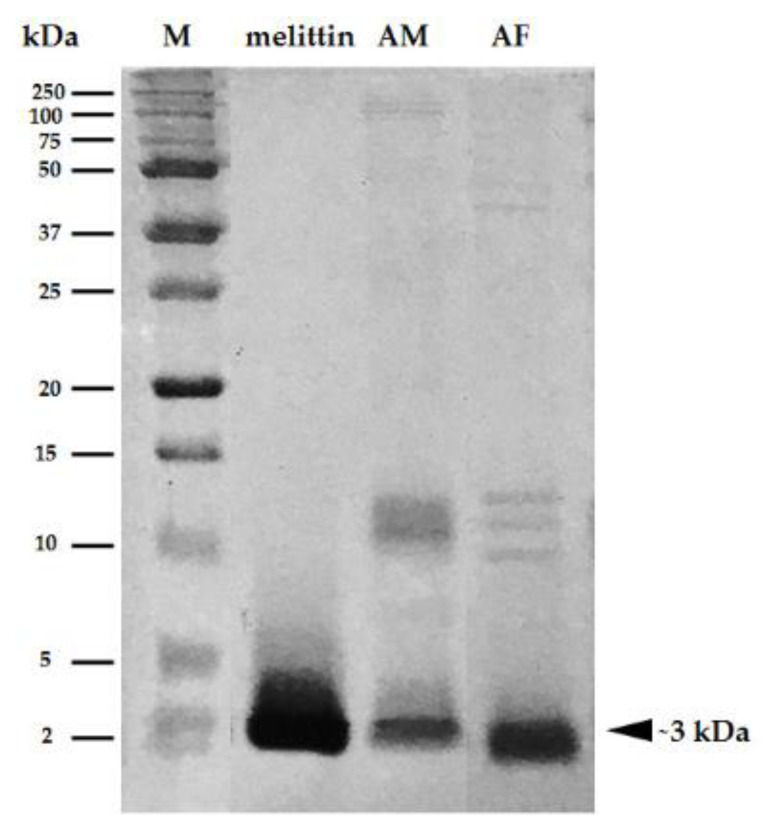
SDS-PAGE analysis of melittin peptide and bee venom extracts obtained from *A. mellifera* (AM) and *A. florea* (AF). Melittin peptide and venom extracts were separated on 15% SDS-PAGE and the patterns of the protein in all samples were visualized by 0.1% Coomassie Blue R250 staining. The major protein component was mainly found at ~3 kDa indicating melittin.

To confirm that melittin is the principal component in the crude venom isolated from *A. florea*, the protein pattern of the bee venom obtained from *A. florea* was studied by SDS-PAGE in comparison to *A. mellifera* and synthetic melittin (*A. mellifera*). Equal amounts of proteins were prepared and separated on 15% SDS-PAGE. The protein patterns of bee venom extracts obtained from *A. florea* were similar to those that were found in the bee venom obtained from *A. mellifera*. The major protein component was mainly found at ~3 kDa, which was identical to both the *A. mellifera* venom extract and melittin (Figure 1). This result clearly demonstrates that melittin was predominantly found in the venom extract that had been isolated from *A. florea*. Therefore, the melittin peptide of *A. florea* (Mel-AF) was synthesized in order to study its molecular mechanism that was involved in anticancer activity.

### 2.2. Anti-Proliferative Effect of Mel-AF on A375 Cells

**Figure 2:**
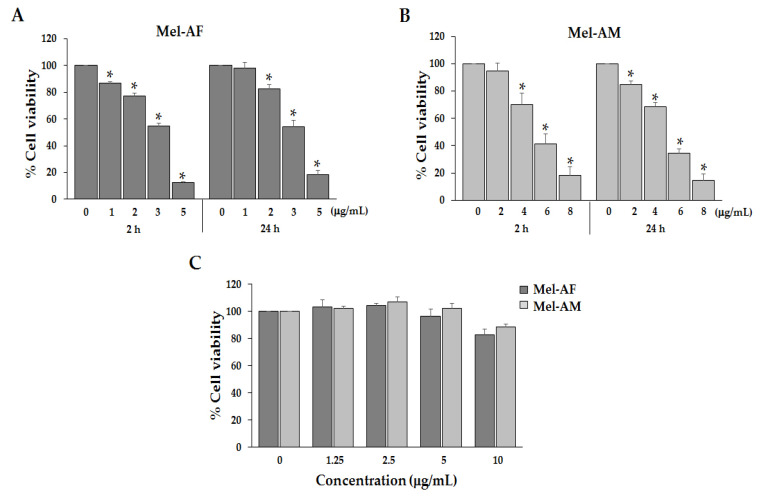
Effect of Mel-AF on A375 cell and NIH-3T3 cell viability. The cells were treated with the indicated concentration of Mel-AF (or Mel-AM) for 2 and 24 h. The viable cells after treatment were determined by MTT assay. (**A**,**B**) Anti-proliferative effects of Mel-AF compared to Mel-AM on A375 cells. (**C**) Non-cytotoxic effects of Mel-AF and Mel-AM on NIH-3T3 cells. Data are presented as mean ± SEM values of three independent experiments that were performed in triplicate. * *p* < 0.05 was determined by one-way analysis of variance.

To determine the inhibitory effect of Mel-AF on the growth of A375 cells, we analyzed cell viability by MTT assay. The cells were treated with several concentrations of Mel-AF (or Mel-AM, doxorubicin (DXR) and 5-fluorouracil (5-FU)) for 24 h. The results demonstrated that Mel-AF, Mel-AM and DXR inhibited cell proliferation of A375 cells in a concentration-dependent manner with IC_50_ values of 3.38, 4.97 and 4.31 µg/mL, respectively (Table 1). After administration with 5-FU, the IC_50_ value was found to be more than 100 µg/mL. This result indicated a degree of potency of Mel-AF against A375 cells at a lower dosage than Mel-AM and anti-cancer drugs. Moreover, treatment with Mel-AF (or Mel-AM) in the range of 1–5 µg/mL (or 2–8 µg/mL), respectively, significantly decreased the cell viability of A375 within 2 h, while a high concentration resulted in cell death by more than 50% (Figure 2A,B).

Moreover, Mel-AF and Mel-AM at concentrations above IC_50_ were also tested in this study. After treatment of the A375 cell for 2 and 24 h by Mel-AF at 5 µg/mL and Mel-AM at 8 µg/mL, cell death was observed more than 80%. These concentrations of melittin were not appropriate for further experiments such as apoptotic gene and protein expression (Figure 2A,B). Thus, the concentrations of Mel-AF that used in the experiments were 1 and 2 µg/mL while concentrations of Mel-AM were 2 and 4 µg/mL. These concentrations were suitable for study of cellular alterations.

Additionally, the cytotoxic effect of Mel-AF was determined in normal cells when normal mouse embryonic fibroblast (NIH-3T3) cells were performed and treated with Mel-AF (or Mel-AM) in the range of 1.25–10 µg/mL for 24 h. The results reveal the non-cytotoxic effect of Mel-AF on NIH-3T3 cells (Figure 2C).

### 2.3. Localization of Melittin Peptide

**Figure 3:**
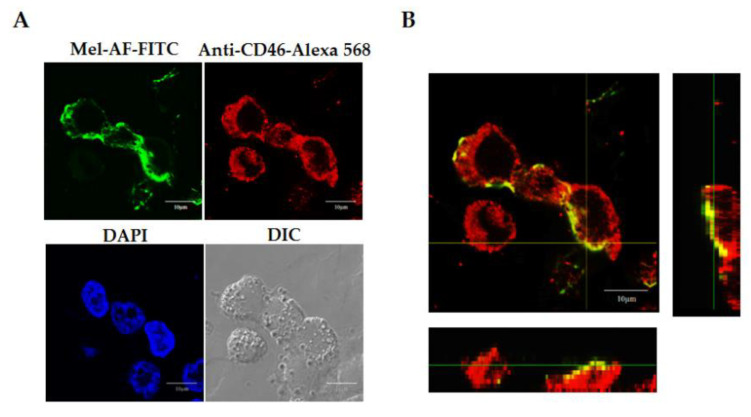
Localization of Mel-AF on A375 cells was analyzed using a confocal laser scanning microscope. The cells were treated with FITC-conjugated Mel-AF for 2 h. (**A**) Confocal images show the localization of Mel-AF traced with FITC (green). The membrane compartment was indicated by staining with a specific antibody to CD46, a membrane cofactor protein followed by Alexa Fluor 568 rabbit anti-mouse IgG (red). The cellular nuclei were counterstained by DAPI (blue). (**B**) Orthogonal imaging analysis was performed to confirm the localization of Mel-AF on the cell membrane (yellow). Scale bar, 10 μm.

To prove the localization of peptides and that Mel-AF (or Mel-AM) was internalized into the A375 cells, the peptides were traced using the FITC labeling peptide. For the staining of CD46, a membrane cofactor protein was developed to represent the cell membrane compartment. After incubation, the FITC-conjugated peptide (green) was located in the same area as Alexa 568-conjugated CD46 (red) (Figure 3A). To ensure that the peptide was placed on the membrane, the orthogonal imaging generated from Z-stack scanning was analyzed. The results showed the co-localization of FITC-conjugated peptide and Alexa 568-conjugated CD46 (yellow) (Figure 3B). This results indicated and confirmed that melittin was located in the cell membrane of the A375 cells.

### 2.4. Apoptosis Induction by Mel-AF

**Figure 4:**
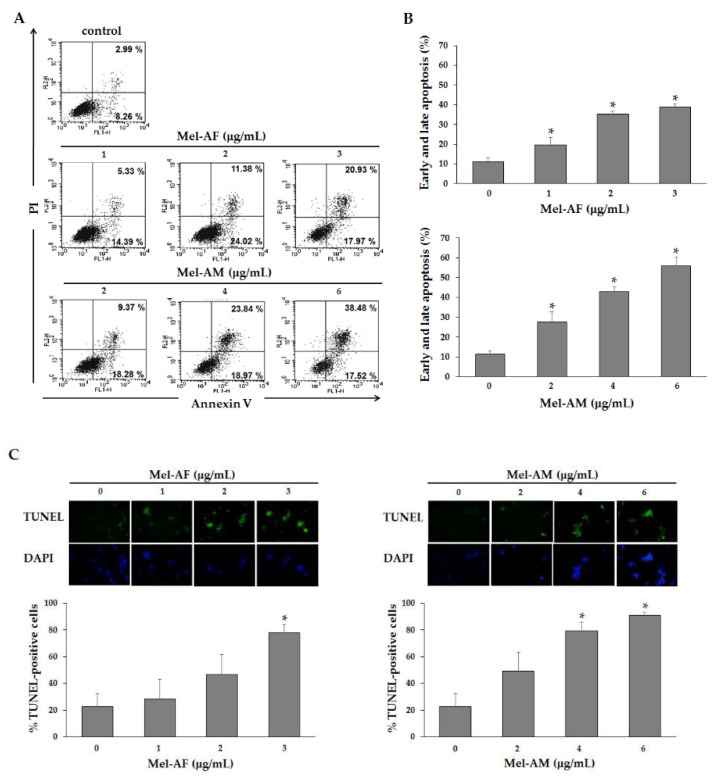
Effect of Mel-AF on apoptosis induction in A375 cells. Cells were treated with the indicated concentrations of Mel-AF (or Mel-AM) for 2 h. (**A**) Cells were then stained with FITC-Annexin V and PI to analyze early apoptotic (Annexin V+ PI−) and late apoptotic/death (Annexin V+ PI+) by flow cytometry. (**B**) Histogram shows the percentages of apoptotic cells for each treated sample at 2 h. (**C**) DNA fragmentation in treated cells were studied by TUNEL assay and analyzed by Flow cytometer. Data are presented as the mean ± SEM values of three independent experiments performed in triplicate. * *p* < 0.05 was determined by one-way analysis of variance.

To investigate whether Mel-AF could induce apoptosis-mediated cell death, phosphatidylserine externalization was examined using an Annexin V-FITC apoptosis detection kit. After 2 h of Mel-AF (or Mel-AM) treatment, the FITC conjugated Annexin V/PI− positive cells were analyzed by flow cytometry. The results demonstrate that Mel-AF (or Mel-AM) triggered early apoptosis (Annexin V+/PI−) when compared with untreated cells. The early apoptosis rates were 14.39%, 24.02% and 17.97% after treatment with 1, 2 and 3 μg/mL of Mel-AF, and the early apoptosis rates were 18.28%, 18.97% and 17.52% after treatment with 2, 4 and 6 μg/mL of Mel-AM, respectively (Figure 4A). The late apoptosis rates were 5.33%, 11.38% and 20.39% after treatment with 1, 2 and 3 μg/mL of Mel-AF, and the late apoptosis rates were 9.37%, 23.84% and 38.48% after treatment with 2, 4 and 6 μg/mL of Mel-AM, respectively (Figure 4A). Early and late apoptosis (Annexin V+/PI− and Annexin V+/PI+) detection showed significant increases in Mel-AF (or Mel-AM) treated cells in a dose-dependent manner (Figure 4B). These outcomes indicated that Mel-AF triggered apoptotic cell death.

Moreover, DNA fragmentation, which is one feature of apoptosis, was detected by TUNEL assay. DNA fragmentation was observed in A375 that had been exposed to Mel-AF (or Mel-AM) using a fluorescence microscope. The results demonstrated that treatment with Mel-AF (or Mel-AM) accrued TUNEL-positive cells. To quantify the DNA fragmentation, Mel-AF (or Mel-AM) treated cells were assessed by flow cytometry analysis. It was found that treatment of Mel-AF (or Mel-AM) elevated the amount of TUNEL-positive cells indicating a significant accumulation of DNA fragmentation at more than 80% in 3 μg/mL of Mel-AF, and 4 and 6 μg/mL of Mel-AM treatment (Figure 4C). Taken together, the above results indicate that Mel-AF was able to effectively induce the apoptosis of A375 cells in a dose-dependent manner.

### 2.5. Activation of Apoptosis-Related Genes and Proteins

**Figure 5:**
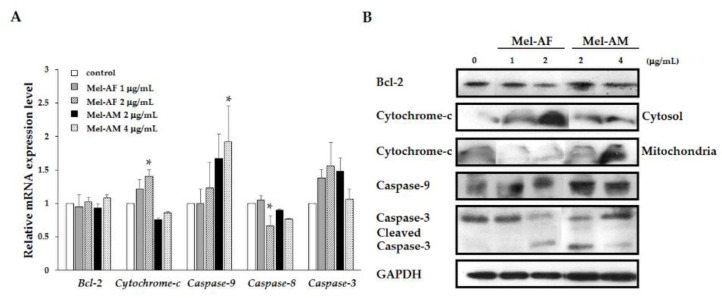
Mel-AF induced apoptosis-related gene and protein expression through the intrinsic mitochondrial pathway in A375 cells. (**A**) Apoptosis-related genes, including Bcl-2, cytochrome-c, caspase-9, -8 and -3, were examined by quantitative real-time PCR. Relative mRNA expression was normalized to GAPDH internal control. (**B**) Western blot analysis of apoptotic protein expression was employed involving Bcl-2, cytochrome-c, caspase-9 and -3 with specific antibodies. GAPDH was used as an internal control to show equal protein loading. Data are presented as mean ± SEM values of three independent experiments performed in triplicate. * *p* < 0.05 was determined by one-way analysis of variance.

Due to the fact that apoptosis was involved in biochemical alterations and that the activation of caspases plays a central role in this mechanism, the possible apoptosis mechanism involved in the Mel-AF (or Mel-AM) treated cells was examined by quantitative real-time RT-PCR. The results revealed the significant increasing of *cytochrome-c* mRNA and elevated trends of *caspase-9* and -*3* mRNA levels in A375 that had been treated with Mel-AF. Mel-AM treated cells had significantly up-regulated of *caspase-9*. Up-regulation of *cytochrome-c* mRNA in Mel-AM treated cells was also not detected. Expression of *Bcl-2* and *caspase-8* mRNA revealed no remarkable alterations in both Mel-AF and Mel-AM treated cells (Figure 5A). Western immunoblotting analysis was performed to strengthen the expression of Bcl-2, cytochrome-c, caspase-9 and caspase-3. Our data demonstrate the translocation of cytochrome-c from mitochondria to the cytosol after Mel-AF administration indicating the translocation of cytochrome-c from mitochondrial membrane to cytoplasm which is a major role during apoptosis. Although Mel-AM treated cells did not show noticeably different expression of cytochrome-c in cytosol and mitochondria. Some cytochrome-c expression can be found in the cytoplasm. On the contrary, the translocation of cytochrome-c from mitochondria to cytosol was not found in the untreated cells. Along with releasing cytochrome-c, the expression of caspase-9 was also increased and the cleavage active form of caspase-3 was detected, whereas the production of Bcl-2 protein was no significant alteration (Figure 5B). As cytochrome-c accumulated in the cytosol compartment, an increase in caspase-9 and active form of caspase-3 occurred without the up-regulation of *caspase-8* mRNA. These outcomes indicate that Mel-AF was responsible for apoptotic cell death in A375 cells through the intrinsic mitochondrial pathway.

### 2.6. Mel-AF Suppressed Migration and Invasion Abilities of A375 Cells by Interfering with F-actin Reorganization and Diminishing Epidermal Growth Factor Receptor (EGFR) Activity

**Figure 6:**
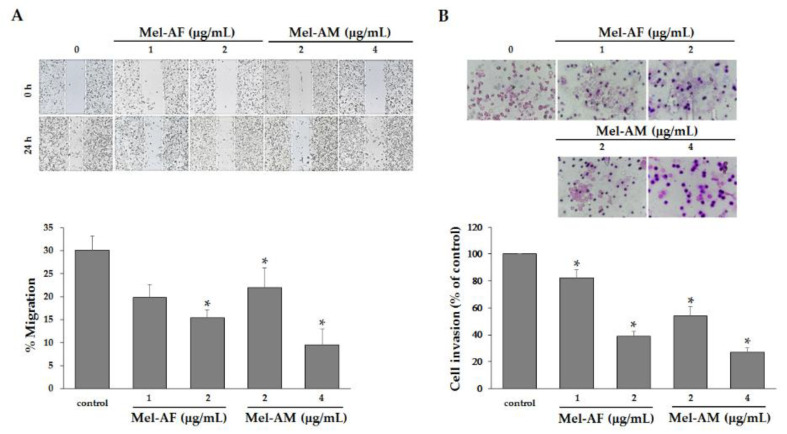
Mel-AF inhibited the migration and invasion activities of A375 cells. (**A**) Suppression of cell migration by Mel-AF was demonstrated using wound healing assay. Images depicting wounds at 0 and 24 h after treatment with Mel-AF (or Mel-AM) are shown (top). The gap distance was measured and the percentage of migration was calculated. (**B**) Decreases in cell invasion were determined by transwell invasion assay when compared with untreated cells (top). The percentage of cell invasion was calculated and compared with 100% cell invasion of the control. Data are presented as mean ± SEM values of three independent trials performed in triplicate. * *p* < 0.05 was determined by one-way analysis of variance.

**Figure 7:**
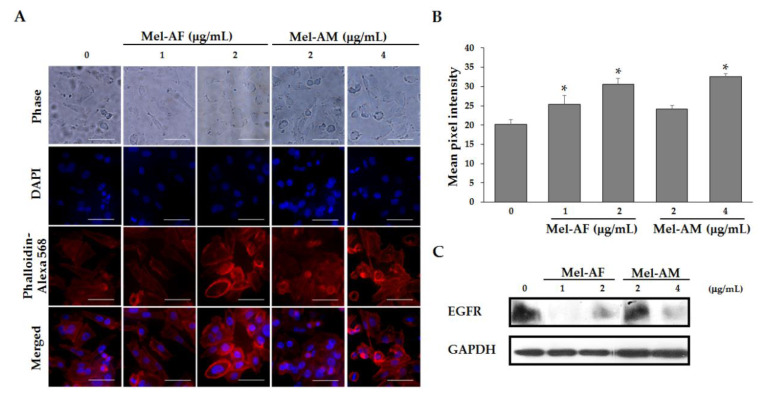
Accumulation of actin stress fibers and suppression of EGFR expression upon Mel-AF treatment for 2 h. (**A**) The treated cells were fixed and the arrangement of actin cytoskeleton was investigated by F-actin staining (red). Images reveal that the cells that were treated with Mel-AF (or Mel-AM) formed abundant amounts of stress fibers beneath the cell membrane when compared with untreated cells. Scale bar, 50 µm. (**B)** F-actin levels were quantified by pixel intensity. (**C**) Expression of EGFR in Mel-AF (or Mel-AM) treated cells was investigated by western blot analysis with specific antibodies. GAPDH was used as an internal control to show equal protein loading. * *p* < 0.05 was determined by one-way analysis of variance.

As migration and invasion are an important process in facilitated the progression and metastasis in cancers, the effect of Mel-AF on the migration and invasion of the A375 cells was investigated by wound-healing and transwell invasion assays. The motility and invasion activities of A375 cells were notably decreased by treatment with Mel-AF (or Mel-AM) in a dose-dependent manner (Figure 6). Because Mel-AF showed anti-motility and anti-invasive activities on A375 cells, we assumed that these effects may have occurred as a result of the influence of Mel-AF on the organization of actin cytoskeleton and the expression of EGFR. Therefore, to determine whether the inhibitory effects of Mel-AF on the migration and invasion of A375 cells were associated with actin polymerization, F-actin staining was performed. In the presence of Mel-AF (or Mel-AM), F-actin stress fibers were obviously thicker, rather stable and reorganized to form stress fibers beneath the cell membrane when compared with the control. This revealed the typical distribution of the actin networks throughout the cells (Figure 7A). The pixel intensity of Phalloidin staining was used to quantify the level of F-actin (Figure 7B). Quantitative accumulation of F-actin was increased in the treatment of Mel-AF (or Mel-AM), which was in accordance with the results found in the fluorescence images. These determinations indicate that Mel-AF promoted the formation of stress fibers and induced actin disorganization.

EGFR signal transduction regulates many cellular activities including migration and invasion and is also implicated in certain cancers. Thus, detection of EGFR expression after Mel-AF was established by western blot analysis. The expression of EGFR was reduced in the presence of Mel-AF (or Mel-AM) when compared with the untreated cells (Figure 7C). These findings confirm that Mel-AF induced defective outcomes in F-actin organization and EGFR signaling resulting in the inhibition of cell migration and invasion.

## 3. Discussion

Bee venom or Apitoxin, a biotoxin produced by the *Apis* species, is a complex mixture of several biologically active substances including enzymes, bioactive amines, peptides and non-peptides. Among these substances, melittin is the principal component in bee venom at approximately 40–50% of the dry weight of the venom [10,14,22]. Moreover, melittin in the bee venom of *Apis mellifera* (European honey bee), a common honey bee found worldwide, has been extensively studied [11,23,24]. On the contrary, *A. florea* (Dwarf honeybee), an indigenous honey bee of southern and southeastern Asia, is poorly understood and has been less studied in terms of its biological properties. Previously published data on melittin obtained from the *Apis* species indicate that melittin was the most prominent component of bee venom at approximately 66 and 76% content for *A. florea* and *A. mellifera*, respectively [13]. In this study, protein analysis by SDS-PAGE revealed that the most abundant protein present in the crude bee venom extract obtained from *A. florea* was 3 kDa, which was identical to that of the melittin and crude extracts obtained from *A. mellifera* (Figure 1). This confirmatory result indicated that the principal protein component in *A. florea* venom was melttin. Melittin is a cationic peptide consisting of 26 amino acids with the chemical formula C_131_H_228_N_38_O_32_, MW 2847.5 Da [14,19]. In a previous study conducted by Maitip and Chantawannakul (2017), the melittin amino acid sequence of *A. florea* was analyzed and submitted to GenBank (GenBank accession number AMP82000.1). It was found that some amino acid sequences of melittin obtained from *A. florea* were different from *A. mellifera* in the way that they exert a range of different activities. In the present study, we have demonstrated that synthetic melittin of *A. florea* (Mel-AF) and *A. mellifera* (Mel-AM) exhibited an anti-proliferative effect on human malignant melanoma (A375) cells in a dose-dependent manner. Noticeably, Mel-AF was used at only half the inhibitory concentration of Mel-AM. In comparison with anti-cancer drugs, Mel-AF also exhibited a comparable effective activity toward doxorubicin (DXR) that was greater than 5-fluorouracil (5-FU). Moreover, the duration of the time of treatment required to inhibit the growth of A375 cells was within 2 h, indicating that Mel-AF and Mel-AM exhibited a rapid killing effect against A375 cells (Figure 2A,B). This observation was in accordance with the findings of previous reports which reported on the growth inhibition and rapid toxicity by *A. mellifera* venom and its melittin within 2 h on human melanoma (A2058) cells [25]. Importantly, the lethal effects induced by Mel-AF (or Mel-AM) was found in A375 cells, but it did not have a cytotoxic effect on mouse normal fibroblast (NIH-3T3) cells (Figure 2C). Likewise, several findings have demonstrated the cytotoxicity of bee venom (*A. mellifera*) on a number of cancer cells, but had an insignificant effect against normal cells such as normal skin fibroblasts, Detroit 551 and normal A10 fibroblast cells [25,26]. These results suggest that Mel-AF and Mel-AM selectively killed the cancer cells in vitro, whereas they displayed low toxicity to normal cells.

The localization of Mel-AF after cellular uptake for 2 h was further investigated. Fluorescent imaging analysis demonstrated that FITC-conjugated Mel-AF (or Mel-AM) to be conformably located in the membrane compartment. The orthogonal image assembled from confocal Z-stack also confirmed the location of these peptides on the cell membrane (Figure 3). This outcome consented to an explanation on the electrostatic interaction of melittin and membrane surfaces [15,27]. Melittin is comprised of positively charged amino acids at the carboxy-terminal region (residues 21–26) which interacts selectively with negatively charged lipids such as phosphatidylglycerol (PG), phosphatidylserine (PS) and phosphatidic acid (PA) [14,16,19,28]. At particular concentrations, this peptide is oriented perpendicularly to form pores in the membrane leading to leakage of the phospholipid bilayers and aggregation of membrane proteins [19,29], whilst higher concentrations of the peptide caused membrane fragmentation [14,16]. With regard to the data, we hypothesized that Mel-AF could bind and incorporate into the cell membrane. These outcomes precede the change of the membrane structure and membrane proteins and the activation of signaling molecules including apoptosis signaling. In addition, the advanced progressive abilities of cancer would be reduced.

The Mel-AF mediated cell death in terms of apoptosis was speculated upon and then further investigated. In the cells undergoing apoptosis, phosphatidylserine (PS) externalization, an early biochemical change, was initiated. The PS on the inner membrane layer flipped out to the outer leaflet of the cell membrane and the externalized PS could be detected through interaction with Annexin V [30]. Our results clearly demonstrate that Mel-AF (or Mel-AM) induced externalized PS (Annexin V+, PI−) representing early-stage apoptosis; however, upon increasing the concentration of Mel-AF (or Mel-AM), the treated cells tended to reach late apoptosis (Annexin V+, PI+) (Figure 4A,B). Taken together, the data demonstrated that Mel-AF (or Mel-AM) significantly increased early and late apoptosis. The following alteration after PS translocation to the cell surface was involved with the DNA fragmentation, a hallmark of apoptosis [6,7]. The fragmented DNA in the A375 cells exposed to Mel-AF (or Mel-AM) was examined by TUNEL assay and the results indicated the appearance of DNA strand breaks that increased in a concentration-dependent manner (Figure 4C). Thus, our study definitively confirmed the potential effect of Mel-AF in apoptosis induction.

As a molecular phenomenon involving apoptosis, *A. mellifera* venom and its melittin have been reported for their anti-cancer effects through several mechanisms that include either the extrinsic death receptor pathway or the intrinsic mitochondrial pathway, which indicate caspase-independence in different types of cancer cells [10,14,15]. For instance, melittin induced apoptosis via death receptors and inhibited the JAK2/STAT3 pathway in ovarian cancer cells [17] and also promoted apoptotic cell death via the mitochondrial signaling pathway, but not via the FAS/FASL pathway in human gastric (SCG-7901) cells [31]. Furthermore, *A. mellifera* venom and melittin also activated apoptotic cell death via a calcium-dependent mechanism in human melanoma (A2058) cells [25].

In this study, the intrinsic mitochondrial mechanisms of apoptosis mediated by Mel-AF in A375 cells was presumed to have occurred as a result of the apoptotic related gene and protein expression. The triggering of apoptosis by Mel-AF (or Mel-AM) involved the activation of cytochrome-c release and caspase-9 expression leading to stimulation of a key executioner caspase-3 and ultimately apoptotic cell death via the intrinsic mitochondrial pathway (Figure 5). Since it has been well studied in terms of programmed cell death (PCD), the intrinsic mitochondrial pathway is initiated in the cell that is responsible for the release of pro-apoptotic molecules, such as cytochrome-c, and results in increased levels of mitochondrial permeability. The translocation of cytochrome-c that normally localizes in the mitochondrial intermembrane space into the cytoplasm is regulated by the Bcl-2 family which consists of two main groups of pro-apoptotic and anti-apoptotic proteins. Bcl-2 is an anti-apoptotic protein that acts by obstruction of cytochrome-c as it is released from mitochondria [6,30]. However, our results indicate an increase of cytochrome-c occurred while no alteration was observed in of Bcl-2 in both the mRNA and protein levels. It is possible that the mitochondrial release of cytochrome-c is promoted by other pro-apoptotic proteins or apoptotic factors, and the function of anti-apoptosis is manipulated by the balance of the pro- and anti-apoptotic proteins. Notably, the release of cytochrome-c in the cytoplasm activates downstream caspase-3 via the formation of an apoptosome complex consisting of cytochrome-c, caspase-9 and other apoptosis proteins to undergo apoptosis [6,30].

In this study, we found that Mel-AF (or Mel-AM) up-regulated both mRNA and protein levels of caspase-9, which is the upstream caspase for intrinsic pathway. In order to determine the upstream caspase for the extrinsic pathway, *caspase-8* was performed to ensure that these processes rely on the intrinsic pathway and not the mediated extrinsic mechanism. The results showed that the mRNA level of *caspase-8* was remarkably decreased in Mel-AF and insignificantly changed in the Mel-AM treated cells. This evidence implies that apoptosis induction by Mel-AF (or Mel-AM) in A375 cells was not involved with the extrinsic pathway. Hence, we have concluded that the administration of Mel-AF enhanced caspases activation and promoted apoptotic cell death through the intrinsic mitochondrial pathway in A375 cells. According to our study, Mel-AF provides an anti-cancer effect by promoting apoptosis and is less pronounced in normal cell toxicity in vitro.

Additionally, in a recent study, we found that Mel-AF (or Mel-AM) suppressed A375 cell migration and invasion abilities in a dose-dependent manner (Figure 6). It is well known that cancer cell motility is a fundamental ability and it is particularly important for its invasion and progressive stage, which requires the organization of actin cytoskeleton and migration of the signaling networks. It has also been reported that the cells that failed to reorganize the F-actin cytoskeleton and were enriched with stress fiber often displayed slower movements [32,33,34]. According to our previous results, a reduction of cell motility and the invasion of A375 cells were demonstrated when treatment was administered with Mel-AF (or Mel-AM). Thus, the arrangement of F-actin cytoskeleton was further investigated by F-actin staining. The results showed that F-actin failed to form an actin network throughout the treated cells and then became stabilized as a result of the presence of stress fibers beneath the cell membrane (Figure 7A). This indicated the influence of Mel-AF (or Mel-AM) on the actin structure. Actin cytoskeleton is essentially involved in many cellular functions such as cell movement, proliferation, signaling system and apoptosis. It has been demonstrated that the suppression of cell motility was associated with actin stress, fiber forming and a reduction of actin reorganization [32,35]. Disruption of actin dynamics by F-actin stabilization also has been reported to facilitate apoptotic cell death via an increase in capase-3 activity [36]. Moreover, the depolymerization of F-actin can also be induced by other toxins such as toxin B from *Clostridium difficile* [37,38] and latrunculin (LatA) from the Red Sea sponge *Negombata magnifica* [39,40].

Since actin cytoskeleton involves many cellular processes, several researchers have endeavored to utilize toxins or other related compounds that target actin cytoskeleton as anti-actin drugs to treat cancers [41,42]. According to our results, we have provided supporting information and introduced a possible property of Mel-AF as an actin cytoskeleton stabilizer for cancer treatment. We have also investigated the expression of the epidermal growth factor receptor (EGFR), a transmembrane tyrosine kinase that belongs to the ErbB family and which plays a crucial role in cell proliferation, differentiation and transformation. Its overexpression is associated with tumor progression and has been found in various tumors including melanoma, whereas it is usually not expressed in normal melanocytes [43,44]. Our study demonstrated that melittin from *A. florea* (Mel-AF) or *A. mellifera* (Mel-AM) played an initial role at the cell membrane and directly inhibited EGFR transmembrane protein expression (Figure 7C). Accordingly, EGFR is known to bind to the F-actin cytoskeleton and this interaction provokes the EGFR signal transduction system [45] and results in the activation of downstream events that are related to cell proliferation, survival, invasion and metastasis [46]. Thus, disorganization of actin cytoskeleton and EGFR reduction by Mel-AF or Mel-AM significantly interfered with and resulted in a defective EGFR signaling system. This findings are also in concordant with a previous work that demonstrated the suppression of cell motility and invasion, and inhibition of actin reorganization by melittin from *A. mellifera* in breast cancer cells after stimulation with epidermal growth factor (EGF), an extracellular protein ligand that binds to EGFR [47]. These strongly supported the role of melittin in disruption of the EGF/EGFR signaling pathway and actin organization. However, further studies are necessary to support this determination.

## 4. Conclusions

**Figure 8:**
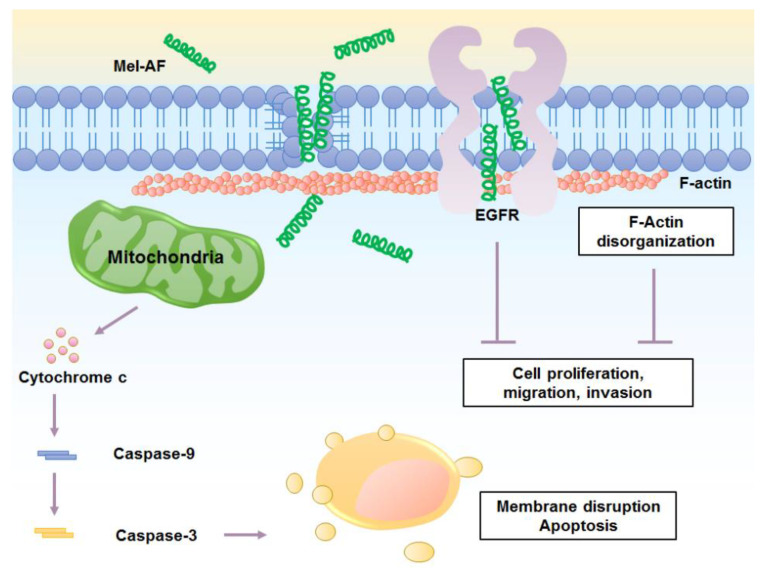
Mechanisms of anticancer activity of Mel-AF in human malignant melanoma cells.

In summary, this current study provides a better understanding of the cellular mechanism of Mel-AF in human malignant melanoma (A375) cells. Our findings indicate the significant potential of Mel-AF in apoptosis induction through activation of the intrinsic mitochondrial pathway. Additionally, it also possesses the capability of disrupting the function of actin cytoskeleton and the EGFR signaling system which then affects the progressive ability of metastatic cancer development (Figure 8). By reason of its effective properties, we have suggested that it could be an attractive candidate as an anti-cancer agent and further applications of Mel-AF should be urgently developed for the enhancement of its potential therapeutic efficacy. However, in vivo studies of its molecular mechanisms and toxicity are still required.

## 5. Materials and Methods

### 5.1. Bee Venom and Synthetic Melittin Peptide

Bee venom extract and the peptide sequence of melittin obtained from *Apis florea* (mel-AF; GIGAILKVLATGLPTLISWIKNKRKQG) and *A. mellifera* (mel-AM; GIGAVLKVLTTGLPALISWIK RKRQQG) were kindly provided by Assoc. Prof. Dr. Panuwan Chantawannakul, Division of Microbiology, Department of Biology, Faculty of Sciences, Chiang Mai University, Chiang Mai, Thailand. The peptide sequence of melittin obtained from *A. florea* had been previously submitted to GenBank (GenBank accession number AMP82000.1). The protein pattern of the crude bee venom extract was analyzed by 15% SDS-PAGE and the gel was stained with 0.1% Coomassie Blue R250. Synthetic high-purity melittin of *A. florea* (Mel-AF) and *A. mellifera* (Mel-AM) were synthesized by Synpeptide (Synpeptide Co., Ltd., Shanghai, China). FITC-conjugated Mel-AF and Mel-AM were manufactured by Genscript (GenScript, Piscataway, NJ, USA). All peptides were stored at −20 °C until being used.

### 5.2. Cell Culture

The human malignant melanoma (A375) cell line was purchased from the American Type Culture Collection (ATCC, Rockville, MD, USA) and the normal embryonic mouse fibroblast (NIH 3T3) cell line was kindly obtained from Dr. Fahsai Kantawong, Division of Clinical Chemistry, Department of Medical Technology, Faculty of Associated Medical Sciences, Chiang Mai University, Chiang Mai, Thailand. These cell lines were cultured in Dulbecco’s modified Eagle’s medium (DMEM) (Gibco, Grand Island, NY, USA) supplemented with 10% fetal bovine serum (FBS), penicillin (100 units/mL) and streptomycin (100 µg/mL) and maintained in a humidified atmosphere of 5% CO_2_ at 37 °C.

### 5.3. Cell Viability Assay

A375 or NIH-3T3 cells (3 × 10^4^ per well) were seeded in a 96-well tissue culture plate and cultured for 24 h. The cells were treated with various concentrations of Mel-AF, Mel-AM and anti-cancer drugs; doxorubicin (DXR) (kindly obtained from Assist. Prof. Dr. Nathupakorn Dechsupa, Department of Radiologic Technology, Faculty of Associated Medical Sciences, Chiang Mai University, Chiang Mai, Thailand) and 5-fluorouracil (5-FU) at the indicated times. The NIH-3T3 was performed as the normal cell control and investigated in terms of its cytotoxicity. MTT assay was used to analyze cell viability. Briefly, 20 µL of 3-(4,5-dimethylthiazol-2-yl)-2,5-diphenyltetrazolium bromide (MTT) solution (5 mg/mL) in the phosphate buffer saline (PBS) was added to the treated cells and they were incubated at 37 °C for 4 h. The supernatant was removed and 200 µL of dimethyl sulfoxide (DMSO) was added to dissolve the formazan crystal that was produced by the living cells. The absorbance was then quantified with a microplate reader using a test wavelength of 540 nm and a reference wavelength of 630 nm. All experiments were performed in triplicate in three independent trials.

### 5.4. Peptide Localization Analysis

A375 cells (2 × 10^5^ per well) were seeded on a poly-L-lysine-precoated sterilized cover glass in a 24-well tissue culture plate for 24 h and treated with FITC-conjugated Mel-AF (or Mel-AM) for 2 h. After being washed with PBS, the cells were then fixed with 4% paraformaldehyde in PBS and permeabilized with 0.1% Triton X-100. Afterwards, the fixed cells were washed in PBS and blocked with 2% non-fat dried milk in PBS at room temperature for 30 min followed by being incubated with mouse anti-CD46 mAb (Beckman Coulter, CA, USA). After being washed, the cells were then incubated with Alexa Fluor 568-conjugated rabbit anti-mouse (Invitrogen, Thermo Fisher Scientific, Waltham, MA, USA). The cellular nuclei were then counterstained by 4′,6-diamidino-2-phenylindole (DAPI) (Invitrogen, Thermo Fisher Scientific, Waltham, MA, USA) and mounted in ProLong Gold antifade reagent (Invitrogen, Thermo Fisher Scientific, Waltham, MA, USA). Images were acquired using a confocal laser scanning microscope (FluoView FV1000, Olympus).

### 5.5. Phosphatidylserine Externalization Analysis

Annexin V-FITC apoptosis detection kit (Calbiochem, Merck, Darmstadt, Germany) was used to assess the externalization of phosphatidylserine induced by Mel-AF (or Mel-AM). In brief, the treated cells were harvested, washed twice with cold PBS and re-suspended in 500 μL of binding buffer. Then, the cells were stained with 1.25 μL of FITC-conjugated Annexin V and incubated at room temperature for 15 min in the dark. After centrifugation, the cells were re-suspended in 500 μL of binding buffer and 10 μL of propidium iodide was added prior to performing flow cytometry analysis.

### 5.6. DNA Fragmentation Analysis

DNA fragmentation was measured using Terminal Deoxynucleotidyl Transferase and Fluorescein-labeled dUTP (TUNEL) assay (Roche, Mannheim, Germany) according to the instructions provided by the manufacturer. Briefly, after 2 h of treatment with Mel-AF (or Mel-AM), the cells were fixed with the fixative solution (4% Paraformaldehyde and 0.1% Triton-X 100 in purified water) for 10 min at room temperature. After the fixation and permeabilization steps, the cells were washed and incubated with the reaction solution (enzyme solution and label solution) for 1 h at 37 °C, in the dark. The cells were then added with nuclei dye and incubated for 5 min at room temperature in the dark. The nuclei dye solution was carefully removed and washed with PBS. Finally, 200 µL of PBS were added to the cells and they were visualized using a fluorescence microscope. TUNEL-positive cells were analyzed as apoptotic cells by flow cytometry.

### 5.7. Apoptosis-Related Genes Analysis

The Mel-AF (or Mel-AM) treated cells were harvested and total RNA was isolated using a TRIzol Reagent (Ambion, CA, USA) according to the manufacturer’s instructions. Total RNA was reverse transcribed into cDNA according to the ReverTra Ace qPCR RT Master Mix (TOYOBO, Osaka, Japan). The amplifications were carried out by SensiFAST SYBR kit (BIOLINE, London, UK) using primer for Bcl-2, Cytochrome-c, Caspase-9, Caspase-8 and Caspase-3. Glyceraldehyde-3-phosphate dehydrogenase (GAPDH) was used as the internal control. The sequences of the primer are listed in Table 2.

The PCR reactions were amplified with initial denaturation at 95 for 2 min, followed by 40 cycles of denaturation at 95 °C for 5 s and annealing/extension at 60 °C for 30 s. The relative fold change in mRNA expression was analyzed using the 2^−ΔΔCT^ method. All experiments were performed in three independent trials.

### 5.8. Determination of Apoptosis-Related Protein Expression

The Mel-AF (or Mel-AM) treated cell lysates were prepared using cold lysis buffer (50 mM Tris-Cl pH 8.0, 0.5 mM EDTA, 150 mM NaCl, 1% Triton X-100, 0.1% SDS) containing protease inhibitors (Merck, Darmstadt, Germany). For determination of cytochrome-c translocation from mitochondria to the cytosol, the cell fractions were separated using the Mitochondria/Cytosol Fractionation kit (BioVision Inc., Milpitas, CA, USA) according to the instructions provided by the manufacturer. An equal amount of protein was separated on a 12 or 15% SDS-PAGE and electroblotted onto a polyvinylidene-fluoride (PVDF) membrane. The immune detection was carried out with the primary antibodies; mouse anti-Bcl-2 mAb (1:500), mouse anti-cytochrome c mAb (1:500), mouse anti-caspase-9 mAb (1:500), anti-caspase-3 mAb (1:500) and rabbit anti-GAPDH pAb (1:2000) purchased from Merck (Darmstadt, Germany). This step was followed by incubation with peroxidase-conjugated goat anti-mouse IgG pAb or anti-rabbit-IgG pAb (Merck, Darmstadt, Germany). Visualization was performed using luminata forte western HRP substrate (Merck, Darmstadt, Germany), and the mixture was immediately exposed to CL-XPosure Film enhanced chemiluminescent (ECL) substrate detection system.

### 5.9. Cell Migration Assay

Cell migration ability of Mel-AF (or Mel-AM) treated cells was measured using the wound healing assay. Cells were seeded (6 × 10^5^ per well) in 6-well tissue culture plates and incubated until they reached 80% confluence. Monolayers were scratched with a 200 μL pipette tip to create a wound and cells were then washed with fresh media to remove the detached cells. The media containing Mel-AF (or Mel-AM) at the indicated concentrations were then added and incubated for 2 h. After replacement with fresh media, cell migration was monitored for 24 h. Migration images were acquired at the reference points and the gap distance was measured [52]. These experiments were performed in three independent trials.

### 5.10. Transwell Invasion Assay

The effect of Mel-AF (or Mel-AM) on the invasion ability of A375 cells was investigated using transwell invasion assay. A375 cells in 200 µL of serum-free medium were plated on the upper chamber of 24-well Transwell inserts with an 8-µm pore size polyethylene terephthalate membrane (BD Biosciences, San Jose, CA, USA) and incubated for 1 h for adherence. The lower chamber was filled with 600 µL culture media containing 10% FBS. Afterwards, the serum-free media containing Mel-AF (or Mel-AM) was then added to the upper chamber for 2 h. After the incubation period, the upper side of the insert was replaced with fresh serum-free medium and incubated for another 24 h. The non-invaded cells in the upper compartment were gently removed with cotton swabs. The cells that passed through the matrigel and were located on the underside of the membrane were determined by Wright–Giemsa staining and counted using a light microscope under a high power field (400×). These experiments were performed in three independent trials.

### 5.11. F-actin Staining

Cells were grown on a poly-L-lysine-precoated sterilized cover glass in a 24-well plate for 24 h. Then, the cells were treated with Mel-AF (or Mel-AM) at the indicated concentration for 2 h. The treated cells were fixed with 4% paraformaldehyde for 10 min at room temperature and washed with PBS. The fixed cells were incubated with 0.1% Triton X-100 for 5 min. After being washed with PBS, cells were incubated with 1% bovine serum albumin for 30 min. F-actin staining was performed with Alexa Fluor 568 phalloidin (Invitrogen, Thermo Fisher Scientific, Waltham, MA, USA) following the manufacturer’s instructions. Images were recorded and pixel intensity of F-actin was analyzed using an inverted fluorescence microscope (Nikon Eclipse T2000).

### 5.12. Expression of Epidermal Growth Factor Receptor (EGFR)

The effect of Mel-AF (or Mel-AM) on EGFR expression was detected by western blot analysis as has been previously described. The protein lysates were separated on 10% SDS-PAGE and electroblotted onto the PVDF membrane. The expression of EGFR was detected by using mouse anti-EGFR mAb (1:500), Santa Cruz Biotechnology (Santa Cruz, CA, USA) (kindly obtained from Assoc. Prof. Dr. Ratchada Cressey, Division of Clinical Chemistry, Department of Medical Technology, Faculty of Associated Medical Sciences, Chiang Mai University, Chiang Mai, Thailand) as the primary antibody and GAPDH was performed as an internal control.

### 5.13. Statistical Analysis

All results are represented as mean ± SEM values. A comparison between groups was determined using one-way analysis of variance. Notably, *p* < 0.05 was considered statistically significant. All calculations were performed using SPSS Statistics 23.0 software (Armonk, NY, USA).

## Figures and Tables

**Table 1:** IC_50_ values of melittin and anticancer drugs on A375 cells.

Compound	IC_50_ (Mean ± SD) μg/mL
Mel-AF	3.38 ± 0.16
Mel-AM	4.97 ± 0.23
Doxorubicin (DXR)	4.31 ± 0.30
5-Fluorouracil (5-FU)	>100

**Table 2:** Sequence of primers used in real-time PCR amplifications.

Primers	Sequences	References
Bcl-2	forward 5′-GTCTGGGAATCGATCTGGAAATCC-3′	[48]
	reverse 5′-TTTGAAACTTCCCAATGAATCAGGAG-3′	
Cytochrome c	forward 5′-GAGCGGGAGTGTTCGTTGT-3′	[49]
	reverse 5′-GTCTGCCCTTTCTTCCTTCT-3′	
Caspase-9	forward 5′-TCAGGCCCCATATGATCG-3′	[48]
	reverse 5′-GACTCCCTCGAGTCTCCAGAT-3′	
Caspase-8	forward 5′-GTGGAGGAAAGCAATCTGTC-3′	[50]
	reverse 5′-TATTAGCCCTGCCTGGTGTCT-3′	
Caspase-3	forward 5′-TGTTTGTGTGCTTCTGAGCC-3′	[48]
	reverse 5′-TCAAGCTTGTCGGCATACTG-3′	
GAPDH	forward 5′-GAAGGTGAAGGTCGGAGTC-3′	[51]
	reverse 5′-GAAGATGGTGATGGGATTTC-3′	
